# Annotations

**Published:** 1903-12-12

**Authors:** 


### ANNOTATIONS.

### The Medical Council and the Royal Colleges.

THE controversy between the General Medical Council and the Royal Colleges of Physicians and Surgeons still continues, and can hardly be said to have been brought any nearer to a conclusion by the Session of the Council which has just terminated. Reports were presented showing that the instruction given to candidates in elementary chemistry, physics, and biology was exceedingly defective, and that the examinations by which the knowledge supposed to be thus acquired was tested were likewise entirely unsatisfactory; but at this point the matter seems to have rested. The Council shrank from invoking the authority of the Privy Council by an official declaration that the instruction and examinations were "insufficient;" and the Royal Colleges, while admitting the insufficiency, were as far as ever from admitting the authority of the Council, with which body, indeed, they declined a conference, although expressing their own intention to remedy the evils which inspection had disclosed. The present deadlock reduces the first stage of medical education to an absurdity, and it is high time that the functions of the Council in relation thereto should be settled by the highest authority. As far as the non-legal mind can determine the question, the Council has been created as a kind of watch-dog, whose business it is to bark if the licensing bodies refuse to do their duty to the public. In a way it may be said to have barked, but it has only barked privately, and, so to speak, in a Pickwickian sense. Even if the Colleges amend the examinations which are complained of, they will do so in almost ostentatious neglect of the watch-dog, and in such a manner as even to deny his right to recall them to a sense of duty. If they allow matters to remain as they are, and if the Council continues to display the masterly inactivity by which its recent proceedings have been characterised, we shall have something like a reproduction of the well-known metrical history of the Walcheren Expedition:—

> "Lord Chatham, with his sabre drawn,
> Stood waiting for Sir Richard Strachan;
> Sir Richard, longing to be at 'em,
> Stood waiting for the Earl of Chatham."

The consequent deadlock is not wholly uninteresting to journalists; but we do not quite see where or how the requirements of the public are provided for.

### The Psychology of Fashion.

IT is a curious fact that in matters of dress it is the man who clothes himself according to reason who is looked upon as mad, while he who would be considered in full possession of his mental faculties must follow without wavering the fantastic dictates of the fashion master of the hour. Although this appears at the first sight to be paradoxical, it is by no means difficult of explanation. The secret of the matter lies in two simple instincts, namely, aversion from strangers and contempt for the weak. The former leads society to adopt a code of signs which is for ever being changed and which is so intricate that a stranger who tries to enter the select circle, be he never so cunning, is sure to betray himself sooner or later. Unfortunately we are not so far removed from savagery but that the power accruing from worldly possessions is still to a large extent the index of social greatness. Intellectual attainments are ever made to subserve material wealth, and he who is poor is weak and accordingly despised. To exclude such folk society again invokes the aid of fashion which insists upon constant changes and more or less extravagant details in dress, so that any particular individual's social worth may be readily gathered from a glance at his outward habiliments. What the exact rules of dress shall be at any one time is immaterial, of course, and being left for decision to the frivolous they are, as might be expected, innocent of any rational features. The man of sufficiently high intelligence to perceive this yet falls into line so far as his purse will allow with his more barbaric and more numerous brothers, for he realises the futility of trying to make a foolish world wise by holding aloof from it. Not till we are educated far above the present level will intellect gain the mastery, and not till then will dress be made subservient to health and comfort and be an index to rather than a concealment of the individual mind.

### The Scarcity of Cod-Liver Oil.

THE present scarcity of cod-liver oil is a matter of serious moment, more especially to patients among the poorer classes. Many of these are quite unable to pay the present famine prices, and some of the hospitals cannot any longer undertake to provide gratuitous supplies. The wholesale price of the Norwegian oil has risen to something like £1 per gallon as compared with the normal price of 2s. 6d. to 3s. The explanation is found in the failure of the cod fisheries as is illustrated by the fall of the number of barrels of oil exported from Bergen from 20,000 in 1901 to 12,000 in 1902, and to less than 4,000 during the present year. It is suggested that the cause of this failure is the increasing number of seals which have during recent years appeared off the Norwegian coast and have driven the fish from their usual feeding and spawning grounds. Measures to cope with this evil have been adopted by the authorities, but not so far with any considerable success. The immediate prospect is not therefore a very hopeful one, and as the Norwegian fisheries have for some time been the main source of the oil it is not probable that the present scarcity will be relieved at an early date. The fishing season commences about the middle of December and extends to April or May, so that it will very shortly be known whether the failure so far as Norway is concerned is or is not likely to be permanent. In any event the discovery of new sources of supply may be anticipated, for the fish doubtless continue to exist and multiply, and it remains only to learn their new habitat. Even then, however, an early fall in prices cannot be expected, as the preparation of the oil involves an expensive process which has in Norway reached a degree little short of perfection. The removal of factories, plant, etc., will mean a considerable outlay, which must needs be reflected in the price of the oil. It is somewhat curious that no really effective substitute for cod-liver oil has been discovered. In spite of recent triumphs, nature's laboratory remains separated by many wide gaps from that of the experimental chemist.

